# Grace Under Pressure: Resiliency of Quality Monitoring of Stroke Care During the Covid-19 Pandemic in Mexico City

**DOI:** 10.3389/fneur.2022.831735

**Published:** 2022-04-06

**Authors:** Raul Medina-Rioja, Gina González-Calderón, Sergio Saldívar-Dávila, Alexander Estrada Saúl, Erika Gayón-Lombardo, Nicole Somerville-Briones, Juan Manuel Calleja-Castillo

**Affiliations:** Neurology Department, National Institute of Neurology and Neurosurgery “Manuel Velasco Suárez”, Mexico City, Mexico

**Keywords:** COVID-19, rtPA, thrombolysis, door-to-needle (DTN) time, acute stroke care

## Abstract

Stroke is one of the leading causes of death and disability among adults worldwide. The World Health Organization (WHO) officially declared a COVID-19 pandemic on March 11, 2020. The first case in Mexico was confirmed in February 2020, subsequently becoming one of the countries most affected by the pandemic. In 2020, The National Institute of Neurology of Mexico started a Quality assurance program for stroke care, consisting of registering, monitoring and feedback of stroke quality measures through the RES-Q platform. We aim to describe changes in the demand for stroke healthcare assistance at the National Institute of Neurology and Neurosurgery during the pandemic and the behavior of stroke quality metrics during the prepandemic and the pandemic periods. For this study, we analyzed data for acute stroke patients registered in the RES-Q platform, in the prepandemic (November 2019 to February 2020) and pandemic (March-December 2020) periods in two groups, one prior to the pandemic. During the pandemic, there was an increase in the total number of assessed acute stroke patients at our hospital, from 474 to 574. The average time from the onset of symptoms to hospital arrival (Onset to Door Time—OTD) for all stroke patients (thrombolyzed and non-thrombolyzed) increased from 9 h (542 min) to 10.3 h (618.3 min) in the pandemic group. A total of 135 acute stroke patients were enrolled in this registry. We found the following results: Patients in both groups were studied with non-contrast computed tomography (NNCT), computed tomography angiography (CTA), magnetic resonance angiography (MRA), digital subtraction angiography (DSA) or more frequently in the pandemic period (early carotid imaging, Holter monitoring) as needed. Treatment for secondary prevention (antihypertensives, antiplatelets, statins) did not differ. Frequency of performing and documenting the performance of NIHSS scale at arrival and early dysphagia test improved. There was an increase in alteplase use from 21 to 42% (*p* = 0.03). There was a decrease in door to needle time (46 vs. 39 min *p* = 0.30). After the implementation of a stroke care protocol and quality monitoring system, acute stroke treatment in our institution has gradually improved, a process that was not thwarted during the COVID-19 pandemic.

## Introduction

Stroke represents one of the leading causes of death and disability among adults worldwide and in Mexico. Prior to the COVID-19 pandemic, the annual incidence of ischemic stroke was 56.4 per 100,000, and the age-dependent prevalence varied between 8 per 1,000 among individuals between 35 and 60 years old and 18 per 1,000 in adults older than 60 years old (1, 2).

In the past 25 years, three therapeutic strategies have significantly impacted stroke outcomes: intravenous thrombolysis, mechanical thrombectomy, and stroke units (3). The implementation of organized systems of stroke care, stroke centers, and standardized quality registries demonstrated efficacy as quality monitoring tools, causing a positive impact on outcomes (4). Among the stroke unit measures thought to impact clinical outcomes are monitoring of time to treatment in reperfusion measures (onset-to-admission and admission-to-treatment time), standardized imaging and clinical assessment, measures to avoid complications, early rehabilitation, and secondary prevention. Continuous monitoring of these performance measures improves the quality of stroke care (4–6). In developing countries, such as Mexico, access to material and human resources is limited, including those essential to acute stroke care (7), and continuous recording and monitoring of quality performance measures is infrequent.

In 2019, a novel beta coronavirus, later named 2019-nCoV (COVID-19), was isolated from three patients in China (8). The World Health Organization (WHO) officially declared a pandemic on March 11, 2020 (9). As the COVID-19 pandemic evolved, a larger frequency of large vessel occlusion acute ischemic stroke was observed among patients with SARS-CoV-2 infection. Several case series have reviewed this association (10). Mount Sinai Health System Hospitals reported that more than half of the LVO stroke patients during New York City's outbreak were COVID-19 positive, and usually younger (11). In another retrospective case-control study, including six New York City hospitals, LVO was present in 31.7% of patients with COVID-19 compared with 15.3% of patients in the control group (12). John and Kesav also reported that 15 out of 20 patients (75%) with COVID-19 and acute ischemic stroke had LVO (13). Also, SARS-CoV-2 positive stroke patients have been found to be younger, with a higher baseline NIHSS (14) and worse clinical outcomes (15–17), including increased mortality (18). These findings suggest that, besides the strain that the pandemic itself causes on health systems, stroke may be particularly aggravated.

The first COVID-19 case in Mexico was confirmed on February 28, 2020, becoming one of the most affected countries by the pandemic (9). After 475 cases were detected, the Secretary of Health of Mexico established a health contingency strategy starting March 24, 2020 (19). A total of 299,759 cases and 35,006 deaths were estimated during the first wave from the beginning of the pandemic until July 2020. In Mexico, during this period, most health institutions directed their attention to patients diagnosed with COVID-19. During this first wave, health services in Mexico City rapidly exceeded their capacity, causing an emerging necessity to transform most hospitals into COVID-19 centers. The demand for attention related to the remaining pathologies among non-COVID-19 centers increased dramatically. The National Institute of Neurology and Neurosurgery (INNN) continued to be one of very few centers still dedicated to neurological care.

In this paper, we aim to describe changes in the demand for ischemic stroke assistance at the National Institute of Neurology and Neurosurgery during the pandemic. Also, to examine whether, even in these circumstances, the implementation of a quality registry with continuous monitoring and feedback would maintain and improve the quality of stroke care, compared to the previous year.

## Materials and Methods

### Acute Stroke Treatment at the National Institute of Neurology

At the Emergency Department of the National Institute of Neurology, patients with suspected acute ischemic stroke are evaluated by a stroke team consisting of at least three neurology residents, an attending neurologist, and a certified nurse. After an initial triage, if the patient has a focal acute neurological deficit, the diagnosis of stroke is suspected, and the patient proceeds to further assessment and management. After initial clinical evaluation, an initial NIHSS score is performed by neurology residents, stroke medicine residents, board certified neurologists and stroke neurologists. Neuroimaging protocols including non-contrast CT and Angio CT are obtained. In candidates for reperfusion therapy during the first 4.5 h after onset, intravenous thrombolysis with IV-rTPA and mechanical thrombectomy is performed (if the patient has LVO). Patients are then hospitalized for subacute care.

During 2019, the National Institute of Neurology started registering acute stroke patients in the RES-Q Stroke registry, and in 2020, a series of actions aimed at improving the quality of stroke care was implemented, including periodic monitoring of stroke care quality measures and feedback whenever a delay or omission was detected.

The Registry of Stroke Care Quality (RES-Q) is an initiative of the European Stroke Organization (ESO) consisting of a worldwide database focused on improving stroke care by monitoring the quality of care and implementing quality standards. The proportion of thrombolyzed cases, door-to-needle time and door-to-groin time are some of the variables registered, and they are continuously monitored and reported (6, 20). In 2020, we started continuous monitoring of quality parameters with immediate feedback using the RES-Q registry. When a protocol breach or an overly extended time occurred, an analysis was performed to find a root cause for the incident, and the process involved in the delay was reviewed.

### Acute Stroke Protocol During the COVID-19 Pandemic

After the pandemic hit Mexico City, the Emergency Department started performing a standardized respiratory triage, classifying patients according to their COVID-19 risk. Patients with respiratory symptoms, oxygen desaturation or unexplained fever were transferred to an isolated “COVID-19 area,” where suspected and confirmed cases were confined. In patients with suspected COVID-19 infection and clinical manifestations compatible with stroke, a non-contract CT scan was performed, and reperfusion therapy, if indicated, was administered in this isolated area (21). If COVID-19 was excluded with a negative PCR test result, the now treated patient was transferred to the general neurology ward.

For this study, we analyzed data for acute stroke patients registered on the RES-Q platform during 2019 and 2020. The patients included were over 17 years old, had an acute ischemic stroke diagnosis (<24 h from onset), and were hospitalized for at least 24 h. Patients younger than 17 years old, non-ischemic stroke (hemorrhagic stroke or transitory ischemic attack), discharged prior 24 h, or whose final diagnosis was different from ischemic stroke were excluded.

We divided the patients into two groups: the “prepandemic” reference group was included from November 1st, 2019, until February 2020, and the “pandemic” group was included from March until December 2020 according to the official dates for the declaration of the epidemic emergency in Mexico City (19). The following variables are recorded and monitored in the registry:

1. Demographic variables: age and sex.
2. Clinical variables: History of prior stroke or transient ischemic attack; active smoking; admission National Institutes of Health Stroke Scale (NIHSS) score; date of stroke determined as date of stroke symptom onset, level of consciousness at admission, and relevant comorbidities.
3. Quality variables: onset-to-door time, door-to-needle time, door-to-groin time, secondary prevention measures (statins, anti-platelets, anticoagulants when appropriate), frequency of NIHSS performed and documented at arrival, and measures to avoid complications (Deep vein thrombosis, pulmonary embolism, aspiration pneumonia).
4. In-hospital treatments: alteplase, mechanical thrombectomy, ICU stay, and mechanical ventilation.
5. Discharge outcomes: mRS at discharge and in-hospital mortality.

### Ethics Committee

The Clinical Research Ethics committee of the National Institute of Neurology and Neurosurgery approved the protocol related to this publication on June 1st, 2021. Protocol number 28/21.

### Statistical Analysis

A univariate analysis was performed using parametric (mean, SD) and non-parametric analyses. Baseline characteristics and outcome variables were compared between both groups using Student's *T*-test for parametric continuous variables, the Mann–Whitney *U* test for non-parametric continuous variables and a chi-squared test for categorical variables.

This study was supported by Boehringer Ingelheim (BI). BI had no role in the design, analysis, or interpretation of the results in this study. BI was given the opportunity to review the manuscript for medical and scientific accuracy as it relates to BI substances, as well as intellectual property considerations.

## Results

During the pandemic, there was an increase in the total number of possible stroke patients evaluated at our hospital: from 474 to 574. After March 2020, over 400 potential stroke patients were suspicious of having COVID-19 on initial triage. A total of 135 acute stroke patients required hospitalization and were enrolled in this registry and divided into two groups: 33 patients were included in the pre-pandemic group and 102 patients were included in the pandemic group. Of the total number of patients included, 69 (51%) were men and 66 (49%) were women. The average age was 63 years (range 19–94).

Regarding stroke severity, there was no clear difference between groups, the mean baseline NIHSS score was 12 in the pre-pandemic group and 14 in the pandemic group (*p* = 0.261). The proportion of patients with altered consciousness was equal among groups (20 patients overall, 5 in the prepandemic group and 15 in the pandemic group, 15%, respectively). No other significant differences among demographic variables between the two groups were observed (Table 1).

**Table 1:** Comparison of baseline characteristics between the prepandemic and the pandemic period.

	**Prepandemic**	**Pandemic**	**Total**	***p***
	***n* = 33 (%)**	***n =* 102 (%)**	***n* (%)**	
Women, *n* (%)	16 (48)	50 (49)	66 (49)	0.957
Average age, y (Range)	63	62	63 (19–96)	
NIHSS performed, *n* (%)	22 (67)	91 (89)	113 (83)	0.002^*^
Average NIHSS	12	14	13.6	0.261
Patients awake at arrival, *n* (%)	22 (67)	78 (76)	102 (76)	0.208
Recurrent stroke, *n* (%)	3 (9)	6 (6)	9 (7)	0.688
Patients who required mechanical ventilation, *n* (%)	5 (15)	9 (9)	14 (10)	0.121
Patients admitted to the ICU, *n* (%)	1 (3)	7 (7)	8 (6)	0.508
Atrial fibrillation detected on admission, *n* (%)	4 (12)	14 (14)	10 (7)	0.814
Carotid stenosis >70%, *n* (%)	0(0)	7 (7)	7 (5)	0.194

* *means statistically significant*.

The systematic recording of NIHSS was significantly different among groups (p.002), it was performed in 22 patients (67%) in the prepandemic group and in 91 patients (89%) in the pandemic group.

During the pandemic, patients sought attention and were rejected in several hospitals prior to arriving at ours. For this reason, the average time from the onset of symptoms to hospital arrival (Onset to Door Time—OTD) for all stroke patients (thrombolyzed and non-thrombolyzed) increased from 9 h (542 min) to 10.3 h (618.3 min) in the pandemic group.

**Figure 1:**
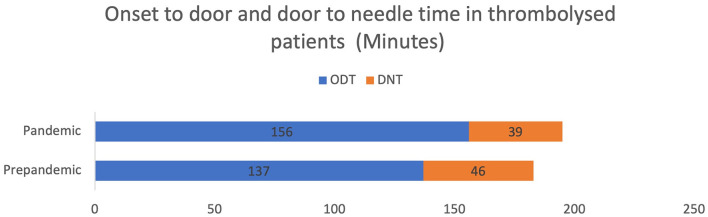
Onset to door and door to needle time.

Even so, the number of patients arriving within the therapeutic window (<4.5 h) improved during the pandemic: 9 of 33 patients (27%) in the pre-pandemic group and 50 of 102 patients in the pandemic group (49%) (*p* = 0.029). This reflected on the treatment strategies offered (Table 2). Twice as many patients were treated with IV alteplase during the pandemic group; 7 (21%) vs. 43 (42%) patients (*p* = 0.03). The registered Door to Needle Time (DNT) decreased from 46 min to 39 min (*p* = 0.30). However, when considering both the OTD and DNT, the Onset to Treatment Time was slightly higher among patients in the pandemic group (Figure 1).

**Table 2:** Comparison of treatment strategies employed between the prepandemic and pandemic period.

	**Prepandemic**	**Pandemic**	**Total**	***p***
	***n* (%)**	***n* (%)**		
Alteplase, *n* (%)^a^	7 (21)	43 (42)	50 (37)	0.03^*^
Door-to-needle time (min)	46	39	42	0.30
Thrombectomy, *n* (%)	4 (12)	18 (18)	22 (16)	0.455

a *All patients treated with alteplase (Alteplase only patients + Alteplase and thrombectomy patients).*

* *means statistically significant*.

The studies performed during the acute phase were as follows: CTA was performed in all patients in both groups. MRA was performed in 1 patient (3%) in the pre-pandemic group and in 1 patient (1%) in the pandemic group. Only 1 patient in the pandemic group had DSA performed. Regarding carotid imaging in the first 7 days, there was a significant increase in the pandemic group (69 vs. 82% *p* = 0.010), and recommendations for Holter monitoring also increased (36 vs. 64%, *p* = 0.004) (Table 3).

**Table 3:** Etiologic work-up.

	**Prepandemic**	**Pandemic**	**Total**	***p***
	***n* (%)**	***n* (%)**		
CTA, *n* (%)	33 (100)	102 (100)	135 (100)	1
MRA, *n* (%)	1 (3)	1 (1)	2 (1.5)	0.397
DSA, *n* (%)	0 (0)	1 (1)	1 (1)	0.569
Carotid Imaging <7 d, *n* (%)	23 (69)	84 (82)	107 (79)	0.010^*^
Holter, *n* (%)	12 (36)	66 (64)	78 (58)	0.004^*^

* *means statistically significant*.

Early rehabilitation is not widely available at our institution, and only 8 patients (6%) in both groups received rehabilitation in the first 72 h of hospitalization. Dysphagia testing markedly increased from 15 to 64% (*p* < 0.000).

Other Stroke Quality Measures that improved during the pandemic were prescription of antihypertensive drugs at discharge (27 vs. 46%, *p* = 0.112), prescription of antiplatelet drugs at discharge (57 vs. 64%, *p* = 0.138), and prescription of statins at discharge (69 vs. 75%, *p* = 0.296) (Table 4). The length of hospital stay was discretely longer in patients from the pre-pandemic group (10.1 vs. 8.9 days, *p* = NS).

**Table 4:** Secondary prevention strategies at discharge.

	**Prepandemic**	**Pandemic**	**Total**	***p***
	***n* (%)**	***n* (%)**		
Antihypertensive medications, *n* (%)	9 (27)	47 (46)	56 (41)	0.112
Antiplatelet drugs, *n* (%)	19 (57)	66 (64)	85 (63)	0.138
Statins, *n* (%)	23 (69)	77 (75)	100 (74)	0.296

A good clinical outcome, defined as mRS ≤ 3, was similar in both groups (45 vs. 54%, *p* = 0.594). Mortality was increased in the pandemic group (3 vs. 10%, *p* = 0.216.), possibly due to the high number of COVID-19 infected patients and the excess mortality in that group.

## Discussion

In this paper, we present the impact of the COVID-19 pandemic on stroke care at the INNN and describe how, despite increased demands, quality monitoring allowed preserved and even improved performance in the usual stroke quality measures.

The pandemic caused by COVID-19 (SARS-CoV-2) caused an increased burden of care in a previously saturated and insufficient health system (22). During this period, most medical resources were focused on patients diagnosed with COVID-19 (SARS- CoV-2) infection. Additionally, it was necessary to design a system where patients were filtered, triaged, and evaluated with protection for all health care personnel and in a specific area. Even though the resources were limited and there was an increase in the demand for attention at the neurologic emergency room, patients in the year prior to the pandemic and during the pandemic year were studied similarly (with NCCT, CTA, MRA, DSA or more frequently (early carotid imaging, Holter monitoring). Treatment for secondary prevention remained stable (antihypertensives, antiplatelets, and statins). Performance and documentation of the NIHSS scale at arrival and early dysphagia test improved, mainly due to the repeated feedback when these measures were not documented.

Regarding reperfusion treatment, the proportion of patients treated with IV thrombolysis increased to 42%, a high percentage by international standards. The door-to-needle time decreased non-significantly in the pandemic period.

A delay of >1 h in reaching the hospital from the onset of stroke symptoms was observed, as patients usually had to visit (and be rejected from) several hospitals before arriving at our institution, due to full occupancy of their nearest ERs. These circumstances led to the referral of patients from one institution to the next before finally receiving medical attention. Another possible explanation could be that patients refrained from seeking immediate medical attention for fear of risking exposure to SARS- CoV-2. These difficulties probably diminished the proportion of patients in our region that received IV thrombolysis.

During the COVID-19 pandemic, it was necessary to organize and adapt “protected stroke codes” where patients were evaluated in protected circumstances (23, 24). At our institution, we followed a protocol that involved an initial triage of symptoms and signs suggestive of COVID-19 infection, a “COVID-19 area” for patients suspected of being infected, and a protocol for the protection of health personnel during the evaluation, transport, and treatment of acute stroke patients. These measures, while necessary, could have delayed the attention of stroke patients with a tangible impact on their ability to respond to reperfusion therapy.

In our study, we observed an increased demand for ER attention and prolonged arrival times due to the reorganization and subsequent collapse of the local health system. The worldwide impact of the pandemic on stroke treatment involved an increase in the number of patients attending emergency rooms (25) but a decrease in the frequency (26, 27) and delayed arrival of stroke patients at the hospital (25, 28) as well as delayed treatment for patients with acute conditions such as AMI (29). It also impacted the frequency of reperfusion therapy, including IV thrombolysis and thrombectomy (30).

Centralization of stroke care in a highly specialized, regional stroke center has been shown to reduce mortality, increase acute interventions and improve outcomes in stroke patients (31).

Other centers have reported similar results to ours. A recent meta-analysis of the stroke hospital care during the pandemic found that patients during the pandemic had a higher probability of undergoing endovascular treatment (32) in another meta-analysis the mortality was also increased during the pandemic time frame (33). This highlights the difficulties encountered for stroke care during the COVID-19 pandemic. Nevertheless, reports from different countries have shown that the existence of organized stroke units diminishes the impact of these unexpected circumstances on the attention of these patients and clinical outcomes (34–36).

Possible advantages of our study are: It is retrospective but based on prospectively collected data with intentional analysis of clinical and quality outcomes in acute stroke. Additionally, in our center, 100% of the patients received were evaluated and treated by neurologists and stroke specialists, followed by the neurovascular clinic from their arrival to the emergency room (ER) to follow-up at the outpatient stroke clinic. Furthermore, all patients had some form of neuroimaging performed; thus, the possibility of an incorrect diagnosis was low.

The main disadvantage is that our study was not powered to detect differences in clinical outcomes and mortality. Most COVID-19 patients were being treated elsewhere and our center prioritized care of non-respiratory neurologic patients. We did not register the COVID-19 status of these stroke patients and thus couldn't analyze nor draw conclusions about its impact on mortality. Also, the time periods and number of patients in each group are not equal and that could limit the direct comparison between both groups. Additionally, this was a single-center study, and extrapolation to other hospitals and countries may be limited.

## Conclusions

The COVID 19 pandemic changed healthcare system protocols and demands. Stroke patients in our country found increased difficulty ability to reach an ED and be appropriately evaluated for acute management.

With the previously described stroke protocol and monitoring system, the quality of acute stroke care in the National Institute of Neurology and Neurosurgery improved. However, there is still need for improvement in the decreasing of DNT, early rehabilitation and reaching a 100% of fulfillment of standardized Stroke Quality Measures.

Among the future goals, we aim to continue to improve through periodic feedback. A major limitation in our center remains the scarce resources for neurologic diseases among the health care budget, which makes thrombolytic and endovascular therapy unavailable for most patients. Information deriving from the stroke registry will help in the optimization of human and material resources.

The establishment of centralized stroke centers with quality-monitored care protocols, the guarantee that these centers will be preserved for cardiovascular and cerebrovascular care, and better organization of the referral system and stroke network, are measures that may secure the quality and sufficiency of stroke care, both in developed and underdeveloped countries.

## Data Availability Statement

The raw data supporting the conclusions of this article will be made available by the authors, without undue reservation.

## Ethics Statement

The studies involving human participants were reviewed and approved by the Clinical Research Ethics Committee of the National Institute of Neurology and Neurosurgery Manuel Velasco Suárez approved the protocol related to this publication on June 1st 2021. Protocol number 28/21. The patients/participants provided their written informed consent to participate in this study.

## Author Contributions

RM-R gathered the information, wrote, and edited the paper. JC-C did the statistical analysis, wrote and edited and the paper. GG-C did the statistical analysis, edited the tables and edited the paper. SS-D did the first draft of the result sections and wrote the first draft of the discussion. EG-L wrote and edited the introduction section. AE wrote and edited the introduction section. NS-B wrote the first draft of the abstract and helped with the statistical analysis. All authors contributed to the article and approved the submitted version.

## Conflict of Interest

The authors declare that the research was conducted in the absence of any commercial or financial relationships that could be construed as a potential conflict of interest.

## Publisher's Note

All claims expressed in this article are solely those of the authors and do not necessarily represent those of their affiliated organizations, or those of the publisher, the editors and the reviewers. Any product that may be evaluated in this article, or claim that may be made by its manufacturer, is not guaranteed or endorsed by the publisher.
